# Clinically relevant orthotopic xenograft models of patient-derived glioblastoma in zebrafish

**DOI:** 10.1242/dmm.049109

**Published:** 2022-04-26

**Authors:** Xiaolin Ai, Zengpanpan Ye, Chaoxin Xiao, Jian Zhong, Joseph J. Lancman, Xuelan Chen, Xiangyu Pan, Yu Yang, Lin Zhou, Xiang Wang, Huashan Shi, Dongmei Zhang, Yuqin Yao, Dan Cao, Chengjian Zhao

**Affiliations:** 1 State Key Laboratory of Biotherapy and Cancer Center, West China Hospital, Sichuan University, and Collaborative Innovation Center for Biotherapy, Chengdu 610041, Sichuan, China; 2 Department of Critical Care Medicine, West China Hospital, Sichuan University, Chengdu 610041, Sichuan, China; 3 Department of Neurosurgery, West China Hospital, Sichuan University, Chengdu 610041, Sichuan, China; 4 Human Genetics Program, Sanford Burnham Prebys Medical Discovery Institute, 10901 North Torrey Pines Road, La Jolla, CA 92037, USA; 5 Department of Gynecology and Obstetrics, West China Second University Hospital, Sichuan University, Chengdu 610041, Sichuan, China; 6 West China School of Public Health, No. 4 West China Teaching Hospital, Sichuan University, Chengdu 610041, Sichuan, China

**Keywords:** Zebrafish, Glioblastoma, Heterogeneity, Blood–brain barrier, PDX, PDOX

## Abstract

An accurate prediction of the intracranial infiltration tendency and drug response of individual glioblastoma (GBM) cells is essential for personalized prognosis and treatment for this disease. However, the clinical utility of mouse patient-derived orthotopic xenograft (PDOX) models remains limited given current technical constraints, including difficulty in generating sufficient sample numbers from small tissue samples and a long latency period for results. To overcome these issues, we established zebrafish GBM xenografts of diverse origin, which can tolerate intracranial engraftment and maintain their unique histological features. Subsequent single-cell RNA-sequencing (scRNA-seq) analysis confirmed significant transcriptional identity to that of invading GBM microtumors observed in the proportionally larger brains of model animals and humans. Endothelial scRNA-seq confirmed that the zebrafish blood–brain barrier is homologous to the mammalian blood–brain barrier. Finally, we established a rapid and efficient zebrafish PDOX (zPDOX) model, which can predict long-term outcomes of GBM patients within 20 days. The zPDOX model provides a novel avenue for precision medicine of GBM, especially for the evaluation of intracranial infiltration tendency and prediction of individual drug sensitivity.

## Abstract (precis)

**Editor's choice:** We established zebrafish glioblastoma (GBM) xenograft models that can be used to perform genetic and biological analysis of GBMs, identify blood–brain barrier-penetrating drugs and predict clinical sensitivity to temozolomide in GBM patients.

## INTRODUCTION

Glioblastoma (GBM) is the most common and lethal primary brain tumor. Despite considerable effort over the past 30 years dedicated to drug development in oncology, more than 90% of novel therapeutics fail in human trials (Hutchinson and Kirk, 2011; Zhou et al., 2020). There are numerous reasons for this high failure rate, the most significant of them being the lack of reliable preclinical models to screen therapeutics prior to use in patients (Kamb, 2005). In fact, many promising drug candidates for GBM have failed during the preclinical phases of development due to the blood–brain barrier (BBB) (Pardridge, 2005). In addition, developing efficient and reliable GBM animal models could enhance the drug discovery process for this disease and would be an especially powerful tool to stratify patient response for personalized medicine. To address this need, patient-derived orthotopic xenograft (PDOX) models have been successfully established in mice, which allow the study of GBM pathogenesis and drug efficacy in the context of a whole animal (Kitange et al., 2009). However, the mouse-based GBM PDOX models are not widely used to evaluate tumor heterogeneity, for ‘co-clinical trials’ or for rapid therapeutic screens. This is because their success rate is highly variable (0-90%), and there is a long latency window following engraftment (2-11 months) (Patrizii et al., 2018; Xu et al., 2018). Therefore, to increase their utility, GBM PDOX models need to produce clinically relevant data faster.

One approach to improve GBM PDOX models is by establishing an alternative or complementary vertebrate model. Zebrafish larvae are one possibility and have advantages that can augment findings from a rodent-based GBM PDOX model, including immune tolerance, transparency for enhanced cellular resolution and the speed at which large numbers of transplants can be performed (Ai et al., 2020; Shi et al., 2020; White et al., 2013). The use of zebrafish as a tumor xenograft model is supported by recent studies confirming that primary human tumors can robustly propagate in zebrafish larvae (Fior et al., 2017; Mercatali et al., 2016). However, the zebrafish-based GBM xenograft model is still not widely utilized in the brain tumor research field (Pudelko et al., 2018; Wehmas et al., 2016). There are several possible reasons for the lack of utilization. First, in previous zebrafish studies, a thorough histopathological characterization of implanted GBM xenografts was lacking, leaving it unclear whether zebrafish GBM xenografts accurately phenocopy the specific intracranial infiltration and vascularization features of the GBM observed in human and rodent models. Second, it is unclear whether a zebrafish GBM xenograft can efficiently identify BBB-penetrating drugs with *in vivo* activity. Therefore, a profound understanding of pathological behaviors of implanted GBM cells and the brain microenvironment of zebrafish larvae is fundamental for establishing the zebrafish model as a reliable human GBM orthotopic xenograft model and using it for therapeutic testing and drug screens.

Here, we began the validation of the zebrafish GBM xenograft model by orthotopically injecting various GBM cells lines that differ in their infiltration and angiogenic properties. We demonstrated that all GBM tumor cells tested can robustly propagate in zebrafish brain and consistently recapitulate their unique pathological characteristics when growing in mammal brains. Next, by using BBB tracers and scRNA-seq, we demonstrated that the BBB in zebrafish larvae is functionally and molecularly homologous to the mammalian BBB. Based on this information, by using primary patient-derived GBM cells, we successfully established a reliable protocol for zebrafish-based GBM PDOX and used it to evaluate the intracranial infiltration tendency and predict long-term individual drug sensitivity. With these advances, we anticipate that the GBM zPDOX model can be widely adopted as a co-clinical test to rapidly define the unique intracranial infiltration potential and therapeutic response of an individual patient's GBM sample. This new zPDOX model should shorten the time required to obtain the preclinical data needed for clinical management of GBM and help further establish precision medicine for GBM as a viable clinical approach.

## RESULTS

### Zebrafish orthotopic xenografts recapitulate the histological features of specific GBM tumors

As the adaptive immune response of zebrafish is not fully established until the end of first month, our previous studies have shown the zebrafish larva to be a versatile host for orthotopic xeno-implantation of human glioma cells (U87MG and U-251MG) (Zeng et al., 2017; Zhao et al., 2018). However, to be considered reliable (or faithful), an orthotopic GBM xenograft model should accurately display the functional heterogeneity that is known to exist both within a tumor and between tumors. To assess the ability of the zebrafish model to reveal GBM heterogeneity, we compared the outcomes of zebrafish xenografts with what has previously been reported in rodent models for three of the most frequently used GBM cell lines (rat C6, human U87MG and mouse GL261).

**Fig. 1.**
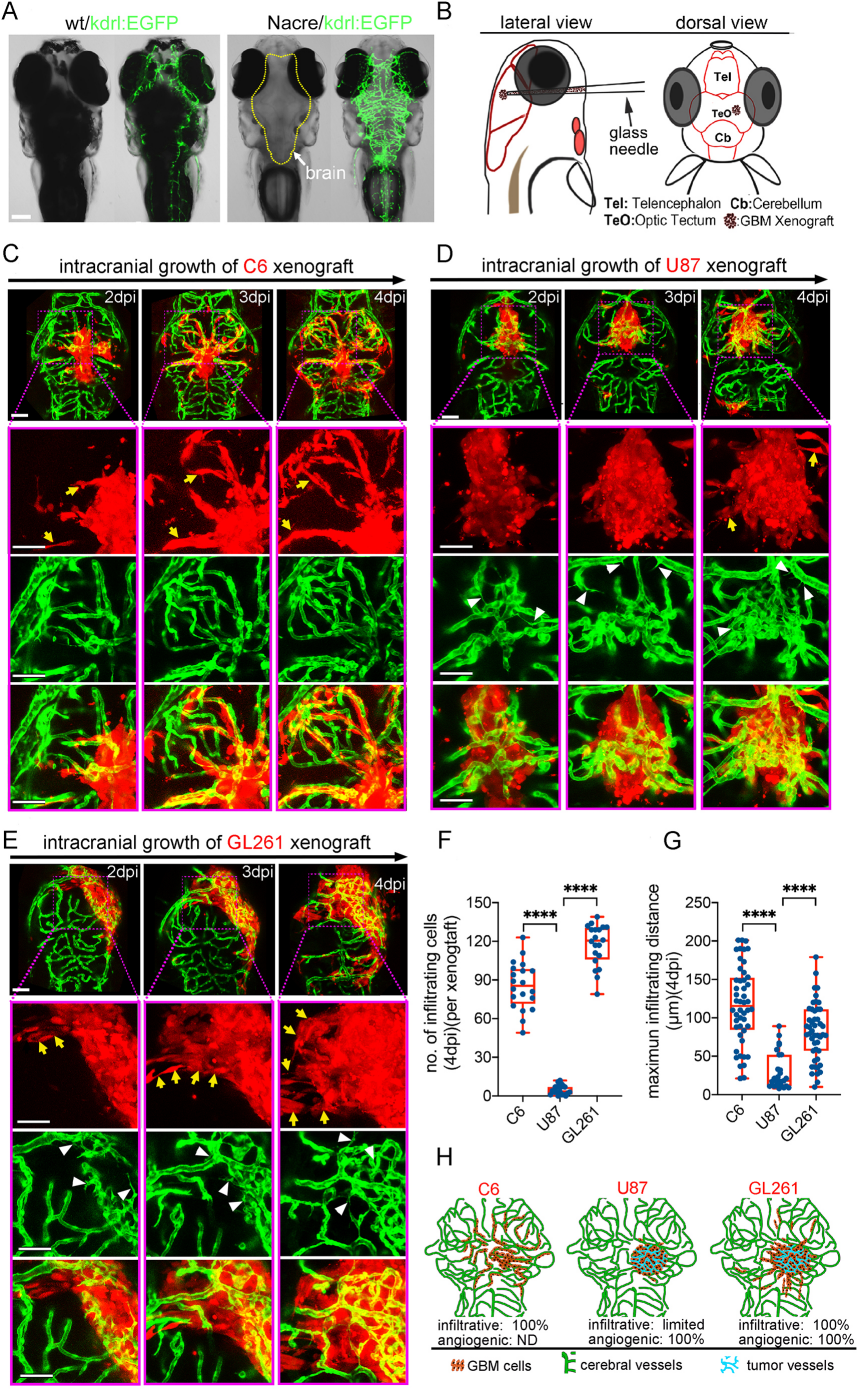
**Zebrafish orthotopic xenografts reveal the specific *in vivo* histopathological features of implanted glioblastoma (GBM) tumor cell lines.** (A) Fluorescence stereomicroscopy of Nacre/*kdrl*:EGFP zebrafish larvae, showing the clear brain vasculature. (B) Strategy of intracranial implantation of glioma cells into zebrafish larvae. (C) Live tracking of rat C6 xenografts shows extremely infiltrative growing pattern (yellow arrows) associating with rare angiogenesis. (D) Live tracking of human U87MG xenografts shows limited intracranial infiltration, but efficient tumor angiogenesis (white arrowheads). (E) Live tracking of mouse GL261 xenografts shows extremely infiltrative growing pattern (yellow arrows) associating with intensive angiogenesis (white arrowheads). (F,G) Box and whisker plots showing the number of infiltrating cells (per xenograft) (F) and maximum infiltrating distance (G) of each identified invading cell from the xenograft edge at 4 days postinjection (dpi). Median (minimum and maximum) values are 85.5 (49-111), 3 (0-12) and 122 (79-139) in F, and 115.5 (21-201), 18 (8-89) and 79 (10-179) in G, for C6, U87MG and GL261, respectively. *n*=6-9 brains were counted for each tested GBM xenograft. (H) Graph showing the progressing patterns of individual GBM xenografts in the zebrafish brains. Scale bars: 100 µm.

To comprehensively track the intracranial invasion and vascularization of GBM xenografts within the zebrafish brain, we employed transparent Nacre/*kdrl*:EGFP zebrafish to visualize all vasculature using fluorescence microscopy (Fig. 1A). By testing implantation at different stages with different cell numbers and volumes, we found that injecting ∼100 GBM tumor cells (Fig. S1) into the optic tectum (TeO) of zebrafish larvae at 3 days postfertilization (dpf) is optimal for GBM orthotopic xenograft (Fig. 1B), yielding a high success rate and enabling a long observation time window (∼10 days) for tumor progression before lethality became significant.

Two days postinjection (dpi), C6 xenografts initiated intensive infiltration along the cerebral capillaries, with some of the invading C6 cells extending pseudopodia and migrating into parenchyma (Fig. 1C; Fig. S2). However, despite extensive infiltration during the first week, tumor neovascularization was never detected in the tumor core of C6 xenografts in zebrafish brain (*n*=0/236). This is similar to the behavior of C6 cells in the brain of syngeneic Wistar rats, in which a diffuse infiltrative growth pattern along with rare neovascularization is observed (Farin et al., 2006; Grobben et al., 2002).

We next tested the human U87MG cell line, which displays a distinct histopathological characteristic from the C6 cells. When orthotopically implanted in immunocompromised mice, U87MG tumor cells propagate as a well-demarcated tumor mass and rarely infiltrate into brain parenchyma. However, like GBM in patients, U87MG tumors are highly vascularized, making it the most commonly used GBM cell type to study GBM angiogenesis and evaluate antiangiogenic therapeutics (Jacobs et al., 2011). Using the same approach for evaluating C6 glioma cells, we tracked the progression of U87MG xenografts in zebrafish brains for 1 week following implantation. From 2 dpi, like the observation in mouse brain, U87MG xenografts initiated intensive angiogenesis and formed complex tumor vasculature within the xenografts (Fig. 1D). However, during this period, the number of U87MG cells invading into the surrounding parenchyma was very limited (Fig. 1F,G).

Finally, we tested the mouse GL261 GBM cell line, which displays many of the same histopathological features of primary human GBM. When implanted into the mouse brain, GL261 tumor cells are extremely infiltrative and induce extensive tumor angiogenesis within the tumor (Baker et al., 2014). As expected, like observations in the brain of syngeneic mice, GL261 xenografts in zebrafish brain displayed infiltrative growth pattern along with hyper-vascularization in the xenograft core (Fig. 1E). Interestingly, compared with C6 and U87MG cells, we found that GL261 cells had distinct intracranial infiltration characteristics depending on their location. At the very front margin of the infiltration, GL261 tumor cells preferred to invade along the host cerebral vessels, while GL261 cells closest to the tumor mass preferentially invaded as cell groups into the parenchyma (Fig. 1F,G). These results indicated high heterogeneity of infiltrating patterns of GBM cells between tumors (Fig. 1H).

Importantly, besides the distinct pathological features displayed by zebrafish orthotopic xenografts compared with the different GBM cell lines, we found that the pathological phenotype within the intracranial xenografts from individual GBM cell lines was extremely stable and homogeneous (Fig. S3). As GBM cell lines are genetically stable and lose their intratumoral heterogeneity after passage cultivation (da Hora et al., 2019), we conclude that the zebrafish orthotopic xenografts faithfully reveal the intratumor heterogeneity between the tested GBM cell lines and the intratumor homogeneity within each tested GBM cell line.

In order to test patient-derived glioma stem cells (GSCs) in our zebrafish models, two GSCs (BNI-21, BNI-23) (Zhai et al., 2020) from Beijing Neurosurgical Institute GSC bank that originally derived from primary GBM patients were tested. These two GSCs were implanted into the zebrafish brain as described previously. Staining of GCSs xenografts with 5-ethynyl-2-deoxyuridine (EdU) at 5 dpi showed the intensive GSC proliferation in zebrafish brain (Fig. S4). Compared with GBM-BNI21, GBM-BNI23 showed a typical infiltrative growth pattern in zebrafish brain, indicating its potential usage in intracranial invasion studies. However, the growth patterns of both tested GCS cell lines within the zebrafish brain were stable and homogeneous, which is similar to the previously tested serum-grown glioma cell lines.

In summary, all tested GBM cells showed their unique infiltrative and angiogenic pathological features when progressing in zebrafish brain (Fig. 1H), matching what has previously been observed in rodent orthotopic models and demonstrating the fidelity of zebrafish orthotopic GBM xenografts in retaining the specific histopathological features of implanted GBM cells.

### scRNA-seq reveals the transcriptional adaption of GBM cells in zebrafish brain

**Fig. 2.**
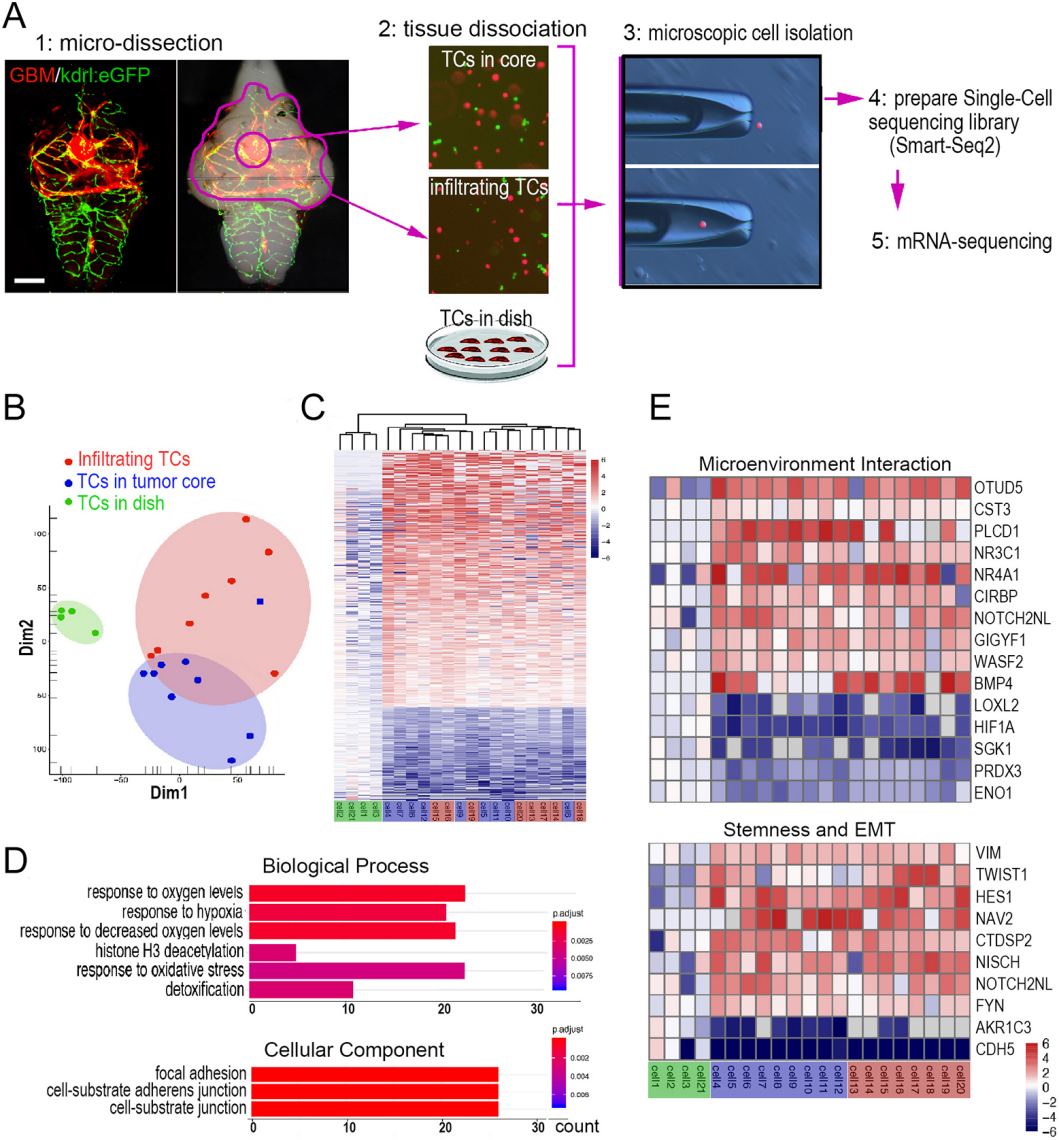
**Single-cell RNA-sequencing (scRNA-seq) reveals the adaptive changes in the transcriptome of GBM xenografts in the zebrafish brain.** (A) Schematic showing the strategy for isolating single GBM-U-251MG-mCherry cells by micromanipulation and preparation of a scRNA-seq library basing on Smart-Seq2 protocol. Scale bar: 100 µm. (B) Principal component analysis (PCA), based on the most variable genes of 21 GBM-U-251MG-mCherry cells, showing distinct patterns of cell clustering. (C) Unsupervised hierarchical cluster analysis, based on all significant differentially expressed genes, showing the similarity of transcriptomes. (D) Gene ontology (GO) pathway enrichment analysis, based on the dysregulated differentially expressed genes between the *in vitro* cultured GBM-U-251MG-mCherry cells and those isolated from xenografts. Count, number of genes related to the enriched GO. The color of the bars denotes the *P*-value. (E) Heatmap based on the differentially expressed genes of cells from xenografts versus *in vitro* cultured cells. TC, tumor cell.

Physiological microenvironment has a significant impact on the transcription of tumor cells, which not only affects tumor progression but also therapeutic outcome (Hutchinson and Kirk, 2011). To investigate the transcriptional changes that may arise, zebrafish were implanted with GBM-U-251MG-mCherry cells, which showed a more typical infiltrative growth pattern in the zebrafish brain and facilitate comparison of transcriptome changes of infiltrating cells in the zebrafish brain. GBM-U-251MG-mCherry cells were allowed to form widespread, tumor invasions for 4 days. At 4 dpi, the core area and tumor invasion area of glioma xenografts were removed. Finally, scRNA-seq was applied to compare the transcriptome of GBM-U-251MG-mCherry cells isolated from zebrafish orthotopic xenografts with that of *in vitro* cultured cells (Fig. 2A).

Principal component analysis (PCA) showed that cultured GBM tumor cells consistently clustered together and apart from the cells isolated from zebrafish brain (Fig. 2B). Within the cluster harboring GBM cells from zebrafish brain, core cells (tumor cells in xenograft core) and infiltrating cells (tumor cells infiltrating in parenchyma) were mixed. Unsupervised hierarchical cluster analysis was also used to computationally compare gene expression profiles of the 21 sequenced cells and gave similar results (Fig. 2C). Gene ontology (GO) analysis was performed to characterize and group all differentially expressed genes between these two populations (adjusted *P*<0.005; upregulated, 346; downregulated, 126) (Table S1). In addition, in zebrafish xenograft-derived GBM cells, significant GO enrichment categories relevant to tumor metastasis were obtained, including cell migration and hypoxia response (Fig. 2D).

Several genes enriched in zebrafish GBM xenografts were highly relevant to epithelial-to-mesenchymal transition and cell migration, including upregulated *VIM*, *TWIST1* and *CTDSP2* (interact with *SNAI1*) and downregulated *CDH5* (Fig. 2E). Within the GBM xenograft cells, we also noticed upregulation of genes that can impact the cerebral microenvironment (Fig. 2E). We suggest that the transcriptional identity of zebrafish GBM xenograft cells is characteristic of GBM tumor cell adaption to the *in vivo* physiological environment of the brain and therefore more likely to resemble invading GBM microtumors observed in the proportionally larger brains of model animals and humans.

### Characterization of the zebrafish BBB and modeling its interaction with GBM xenografts

**Fig. 3.**
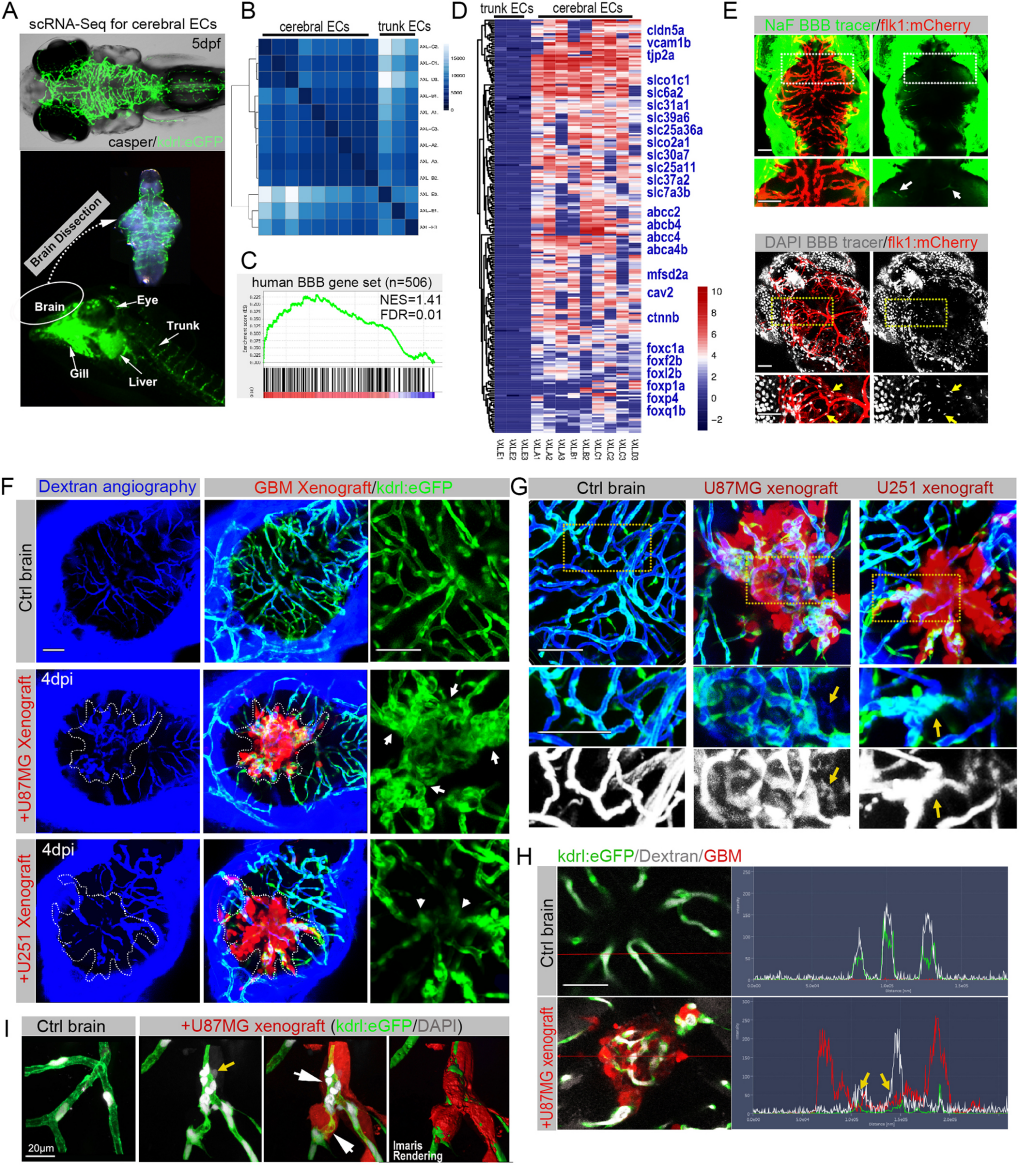
**The zebrafish GBM xenograft provides a visual readout for the structural and functional changes of the tumor-associated blood–brain barrier (BBB).** (A) Schematic showing the strategy of separating cerebral endothelial cells (ECs) from the trunk ECs of zebrafish larvae. (B) Unsupervised clustering analysis based on all significant differentially expressed genes of 12 ECs. (C) Gene set enrichment analysis (GSEA) showing the enrichment of BBB genes in zebrafish cerebral ECs. The BBB gene set contains 506 genes, including tight junction-related genes, solute carrier transporters and ATP-binding cassette transporters. (D) Heatmap showing the enriched BBB genes in zebrafish cerebral ECs compared with the trunk ECs. The bar is log2 scaled. (E) Live fluorescence microscopy of 5 days postfertilization (dpf) zebrafish heads, showing the incapability of small-molecule tracers NaF (376 Da) and DAPI (350 Da) to penetrate the brain parenchyma. Arrows indicate the BBB tracers that are restricted to cerebral capillaries. Areas in the dotted line boxes are magnified below. (F) Fluorescence microscopy of zebrafish cerebral angiography using Dextran Blue (10,000 Da), showing the intensive angiogenesis (arrows) in U87MG xenograft and vascular degeneration (arrowheads) in U-251MG xenograft. Dotted lines indicate the margin of xenografts. (G) High-resolution confocal images of cerebral angiography using Dextran Blue, showing the leakiness (yellow arrows) of tumor vessels in GBM xenografts. Areas in dotted line boxes are magnified below. (H) Fluorescence spectrums of blood vessels with Dextran Blue (white signaling) in control brain or GBM xenograft, showing expansion of Dextran Blue signaling (yellow arrows) beyond the vessel boundaries (green signaling) within the xenografts (red signaling). (I) High-resolution confocal images of DAPI staining, showing the limited leakage of DAPI (yellow arrow) from blood vessels with infiltrating tumor cells (white arrows). Scale bars: 100 µm (E-H), 20 μm (I).

To develop effective GBM drugs, it will be critical to design and evaluate modalities that can circumvent or overcome the BBB associated with GBM cells. To molecularly characterize the BBB in zebrafish larvae, we began our investigation by using scRNA-seq to characterize the transcriptomes of endothelial cells (ECs) isolated from the brain and from the body trunk of zebrafish larvae (5 dpf) (Fig. 3A). Gene expression profile hierarchical cluster analysis highlighted the differential transcriptomes between cerebral ECs and trunk ECs (Fig. 3B). Using gene set enrichment analysis (GSEA), we then compared the transcriptome of zebrafish cerebral ECs to a set of BBB genes previously identified in humans (Qian et al., 2017) (Table S2). GSEA revealed that most of the BBB-related genes were specifically enriched in the zebrafish cerebral ECs compared with trunk ECs (Fig. 3C). We also observed some previously identified cerebral endothelial genes that are enriched in zebrafish cerebral ECs (*foxc1a* and *foxf2b*) along with *ctnnb* (also known as *ctnnb1*; encodes β-catenin), a factor recently proven to be essential in developing and maintaining BBB integrity (Wang et al., 2019) (Fig. 3D).

Next, to functionally test the BBB in zebrafish larvae, the BBB tracers were injected 1-2 h before imaging at 5 dpf. Within 1 h following tracer injection, most cells in tissue outside the brain displayed blue nuclear staining [4′,6-diamidino-2-phenylindole (DAPI)] and green cytoplasmic fluorescence (NaF), suggesting free diffusion of tracers. In contrast, in the brain of zebrafish larvae, both tracers were strictly retained in the cerebral vessels, indicating the existence of a functional BBB in the brain of zebrafish larvae (Fig. 3E).

We next sought to characterize the interaction between GBM cells and the BBB in zebrafish brain at 4 dpi (Fig. 3F). Interestingly, the low-molecular-mass dextran tracer was still mainly restricted in tumor vessels, indicating that the tumor-associated BBB was not functionally destroyed (Fig. 3F, left column). Next, we used more sensitive assays, including high-resolution confocal intravital imaging (Fig. 3G) and quantitative diffusion analysis (Fig. 3H), to more carefully characterize the BBB function within the GBM xenografts. Surprisingly, the more sensitive imaging and functional analysis revealed the limited leakage of Dextran Blue and DAPI from the cerebral vessels in the xenografts (Fig. 3G-I). These results indicate that GBM microtumor/xenografts in zebrafish brain, whether angiogenic or infiltrative, do not simply destroy the zebrafish BBB, but instead initiate slow and steady local damage to BBB organization and function, which are consistent with previous investigation in rodent brain (Watkins et al., 2014). In summary, we demonstrate that the zebrafish GBM xenograft model provides a detailed visual readout of structural/functional changes to the BBB in the presence of GBM microtumors/xenografts.

### Using zebrafish orthotopic GBM xenografts to identify BBB-penetrating drugs

**Fig. 4.**
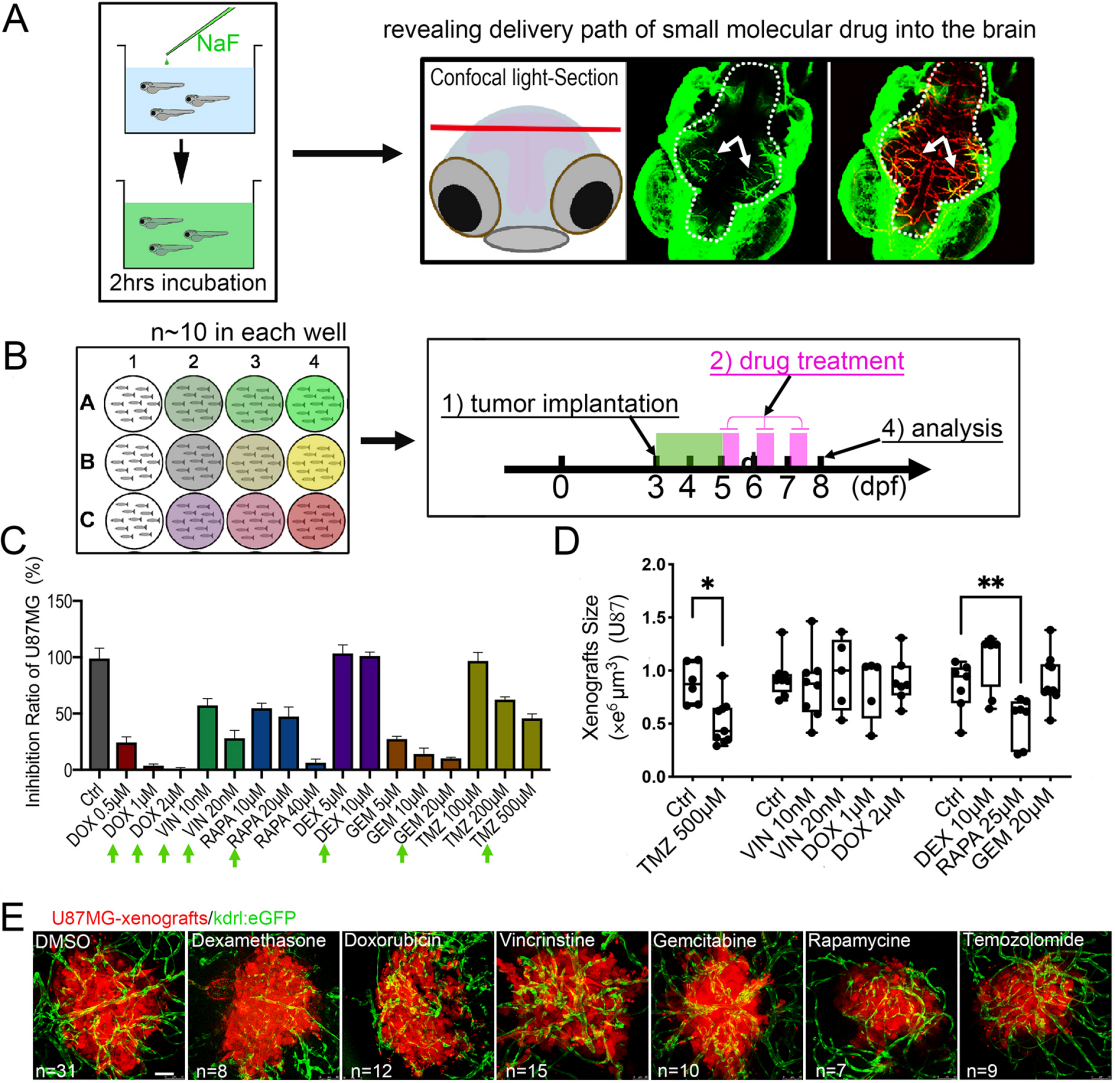
**Zebrafish GBM xenografts enable identification of BBB-penetrating drugs with *in vivo* activity on the tested GBM.** (A) Schematic showing the small-molecule chemical delivery path into the zebrafish brain (5 dpf) from culturing water. NaF (10 μM) was added directly to fish water 2 h before imaging. Within the brain, NaF was restricted to cerebral vessels (arrows), but not freely diffusing in the parenchyma. (B) Schematic showing the strategy of grouping and drug treatment using the zebrafish GBM xenografts (U87MG). (C) Graph showing the growth inhibition of U87MG *in vitro* after 3 days’ treatment by various drugs at different concentrations. Cell number was tested by MTT assay, and the concentrations further tested *in vivo* were marked. (D) Box and whisker plot showing the xenograft size in each group after the drug treatment, showing significant inhibition by TMZ and RAPA. Median (minimum and maximum) values are 0.8725 (0.67-1.1), 0.43 (0.29-0.95), 0.91 (0.719-1.36), 0.8765 (0.415-1.465), 1 (0.532-1.365), 1.032 (0.385-1.045), 0.865 (0.618-1.308), 0.945 (0.413-1.083), 1.247 (0.639-1.298), 0.624 (0.2116-0.731) and 0.816 (0.531-1.381) for control, TMZ 500 μM, control, VIN 10 nM, VIN 20 nM, DOX 1 μM, DOX 2 μM, control, DEC 10 μM, RAPA 25 μM and GEM 20 μM, respectively. (E) Representative images of intracranial xenografts (U87MG) after drug treatment. The numbers of evaluated samples are indicated. Scale bars: 50 µm. Ctrl, control; RAPA, rapamycin; TMZ, temozolomide; VIN, vincristine.

Given the resolution and speed at which interactions between GBM xenografts and the BBB can be modeled in zebrafish larvae, we next evaluated the zebrafish GBM xenografts in screening drugs that are potentially able to penetrate the BBB and inhibit GBM tumor cell growth *in vivo*. It is necessary to first examine whether small-molecule chemicals placed in water can enter brain tissue by simple diffusion. To rule out the possibility, by adding in the water, we tested two florescent BBB tracers: NaF and DAPI. We found that the zebrafish skin tissue outside the brain and the muscle cells in the trunk can be efficiently labeled with green NaF and blue DAPI in the nucleus, indicating that the tracers have penetrated most tissue of the zebrafish (Fig. 4A; Fig. S5). The exception is the parenchymal cells of the brain; they are not labeled at all, indicating that NaF and DAPI have not penetrated the brain parenchyma. However, we found that the cerebral capillaries are highlighted by NaF and the endothelial nuclei are labeled by DAPI, indicating that the tracers are delivered into the brain vessels by blood circulation, but unable to cross the boundaries of brain vessels or the BBB. These results suggest that adding small molecules directly to the culture water is a simple, reliable way to test BBB-penetrating drug candidates in the zebrafish model system.

To test whether zebrafish GBM xenografts can efficiently identify BBB-penetrating drugs with *in vivo* activity, we tested six drugs that were previously evaluated for glioma treatment *in vitro* and *in vivo* (Arcella et al., 2013; Bastiancich et al., 2018; Kellie et al., 2004; Kikuchi et al., 2008; Ostermann et al., 2004). The testing procedure was standardized prior to experimentation (Fig. 4B). Overall, we found that all drugs except for dexamethasone (DEX) were able to inhibit growth of U87MG *in vitro* by 50-100% (Fig. 4C). *In vivo*, only rapamycin and temozolomide (TMZ) were able to significantly repress the growth of GBM xenografts (Fig. 4D,E). Rapamycin has been demonstrated to improve survival of tumor-bearing mice (Arcella et al., 2013), and TMZ is a U.S. Food and Drug Administration (FDA)-approved chemotherapy agent for treating high-grade glioma (Ostermann et al., 2004). The failure of DEX to inhibit the growth of U87MG cells *in vitro* or *in vivo* is consistent with its known function in managing vasogenic edema by reducing the permeability of cerebral vessels (Fig. 4D,E). Doxorubicin, vincrintine and gemcitabine inhibit U87MG cells *in vitro* by up to ∼100%, but had no detectable effect on GBM xenografts *in vivo* (Fig. 4D,E). Previous studies have indicated their incapability or inefficiency in crossing the BBB (Bastiancich et al., 2018; Kellie et al., 2004; Kikuchi et al., 2008). These results indicate the ability of zebrafish GBM xenografts as an *in vivo* model to identify BBB-penetrating antitumor drugs.

### zPDOXs reveal the intratumor heterogeneity of GBM cells from an individual GBM patient

**Fig. 5.**
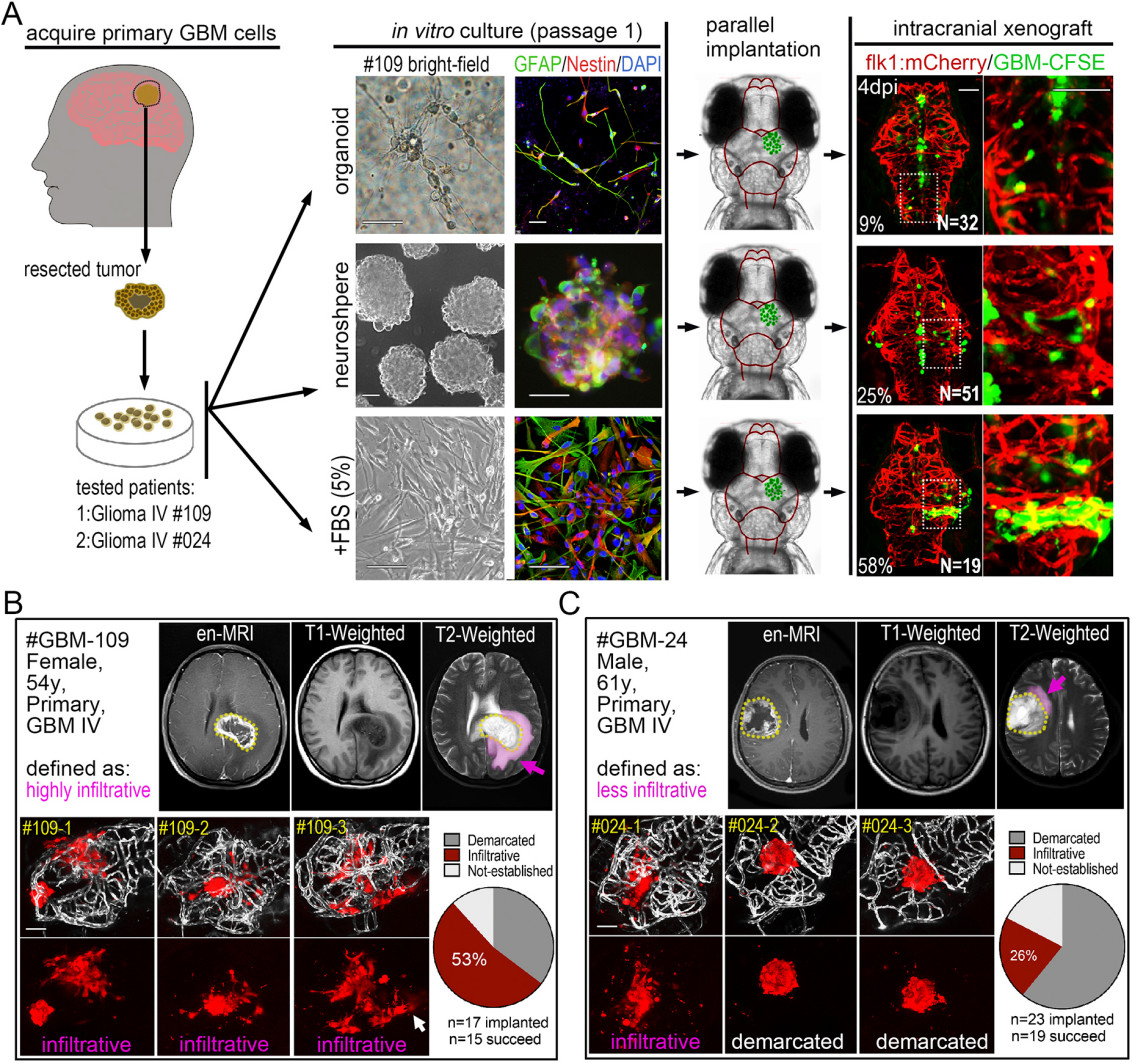
**Orthotopic engraftment of patient-derived GBM cells into the zebrafish brain.** (A) Left: schematic showing the acquisition of primary GBM cells from patients. Middle: bright-field and fluorescent microscopy of *in vitro* cultured primary GBM cells (passage 1) from the same patient as 3D organoid, neurosphere and attached single-layer cells. Immunofluorescence staining showing NES and GFAP expression in differently cultured primary GBM cells. Right: fluorescence microscopy of 4 dpi GBM xenografts from primary GBM cells (passage 1). Primary GBM cells were labeled with CFSE before injecting into zebrafish brain. Two patients (GBM#109 Grade IV and GBM#24 Grade IV) were tested. (B,C) Fluorescence microscopy of 4 dpi patient-derived GBM xenografts from GBM patients (GBM#109, B; GBM#24, C), showing the heterogeneous phenotypes (infiltrative or demarcated) of primary GBM xenografts. Top: #GBM109 was defined as highly infiltrative and #GMB24 was defined as less infiltrative by MRI. Pink labeling (pink arrows) in MRI images indicates the edema zone and potential infiltrating areas. Bottom: brain vasculature is highlighted by fake gray color (*kdrl*:eGFP). Xenografts with more than five individual infiltrating cell clusters (white arrow) were defined as infiltrative, otherwise they were defined as demarcated. The numbers of xenografts with different phenotypes were counted (pie charts). Scale bars: 100 µm.

To expand the zebrafish GBM model, we next sought to generate PDOXs from GBM patients. We first tried to establish GBM xenografts by directly injecting fresh GBM cell suspensions from two surgically resected patient samples (GBM#109 and GBM#24). Unfortunately, both failed to propagate in the zebrafish brain and died within 3 days. To circumvent this issue, we applied a short-term culture of the primary GBM cells prior to injection. For each sample, we tested three culturing methods, including the glioma organoid derivation procedure, the classical neurosphere enrichment method and the attached culturing procedure with serum. For the first generation, primary GBM cells grew robustly in all three culture systems and were enriched with GFAP^+^ and NES^+^ GBM cells (Fig. 5A). Compared with organoid and neurosphere cultured cells, attached culture GBM cells yielded the highest success rate, 58% (attached cells) versus 25% (neurosphere) and 9% (organoid) (Fig. 5A, right panel). The differentiation of primary GBM cells was evidenced by the low incidence of NES^+^ cell ratio and the presence of very long pseudopodia and synapse-like structures in the 3D Matrigel (Fig. 5A; Fig. S7). Compared with the organoid and neurosphere culture methods, although serum-containing medium did not result in the highest NES^+^ cell ratio, it enriched the most tumor cells during a short-term *in vitro* culturing (∼7-10 days) (Fig. S7), which, in our setting, is important to produce robust xenografts in zebrafish brain.

Using the lentivirus-mCherry labeling, we further tested passage 2 cells of these two GBM patient samples. These two GBM samples were originally characterized by a radiologist and based primarily on the size of the edematous zone using postcontrast magnetic resonance imaging (MRI) (Yamahara et al., 2010) (Fig. 5B,C, pink labeling/arrows). GBM#109 was defined as highly invasive and GBM#24 was defined as less invasive. After labeling with lentivirus-mCherry, GBM cells from each patient were implanted into the zebrafish brain, and, by 4 dpi, robust xenografts were produced in most of the samples (*n*=15/17 for GBM#109; *n*=19/23 for GBM#24). Surprisingly, in contrast to the remarkably consistent xenograft phenotype from GBM cell lines, the xenograft phenotypes of patient-derived cells varied, ranging from infiltrative to demarcated (Fig. 5B). Considering the small number of primary GBM cells that were initially implanted in each zebrafish brain, we suspect that the phenotype variation of patient-derived xenografts may reflect the well-characterized intratumor heterogeneity of GBMs within individual patient tumors. To quantify the observed variability, we scored and calculated the percentage of infiltrative and demarcated phenotypes in each patient's xenografts. We found that, even though there was variability, one phenotype predominated: GBM#109 xenografts were 53% infiltrative, while GBM#24 xenografts were 26% infiltrative (Fig. 5B,C; Fig. S8). Ultimately, the predominant phenotype matched the GBM phenotype from each respective patient's MRI.

In summary, we have established a stable method for establishing a zPDOX model of GBM. Our zPDOX procedure can efficiently produce multiple xenografts (dozens) from individual patients within 20 days. Critically, the primary histopathological feature of an individual GBM sample can be ascertained by scoring and tallying phenotypes from multiple samples. These encouraging results raise the possibility of using zPDOX for co-clinical drug selection and patient-specific treatments.

### Comparison of short-term TMZ response in zPDOX with long-term prognosis of corresponding TMZ-treated GBM patients

**Fig. 6.**
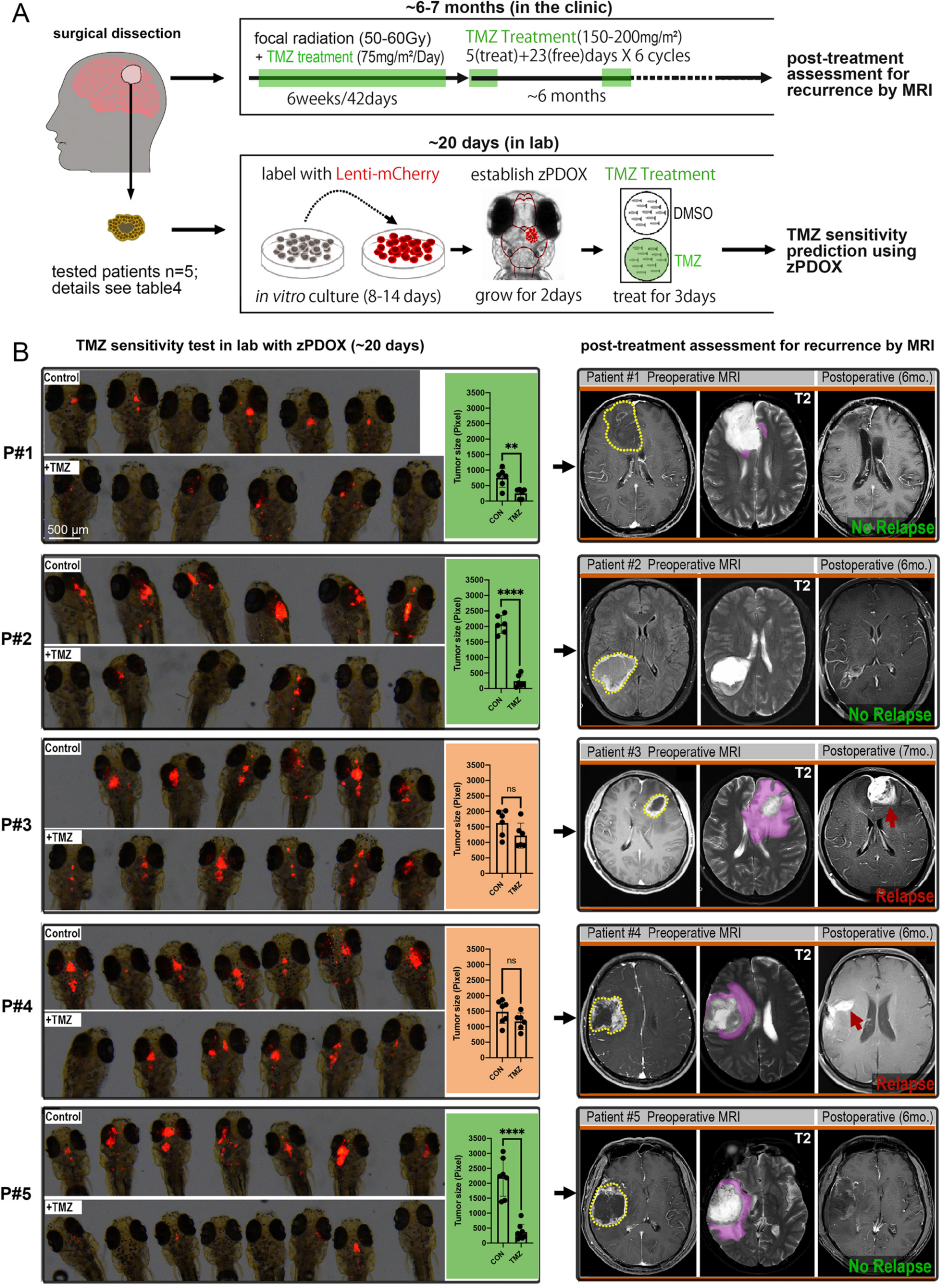
**Short-term TMZ response in zebrafish patient-derived orthotopic xenografts (zPDOXs) predicts the tumor relapse in GBM patients treated with TMZ.** (A) Schematic showing the procedure of clinical treatment of individual GBM patients and the parallel zPDOX operation in the laboratory. Right, top: after surgical dissection of the primary GBM tissue, the patient was subjected to standard radiotherapy and TMZ chemotherapy. Right, bottom: dissected GBM tissue was sent to the laboratory for establishment of the zPDOX and TMZ sensitivity test. (B) Comparison of short-term TMZ response in zPDOX in terms of tumor size inhibition ratio and the effect of TMZ on the corresponding patient in terms of tumor relapse (6-7 months later). Zebrafish recipients with zPDOX were imaged by a fluorescence stereomicroscope, and each xenograft was then scanned by a confocal microscope and measured by ImageJ. Graphs show the inhibition ratio of xenografts in terms of tumor size after 3 days' TMZ treatment. ns, not significant; **P<0.01, ****P<0.0001 (unpaired two-tailed Student's *t*-test). All five GBM patients were examined before surgery (Preoperative MRI) and ∼6-7 months after surgery (Postoperative) by MRI. Dotted line circles, gadolinium-enhanced tumor; pink labeling/arrows, vasogenic edema. Red arrows indicate tumor relapse. Scale bars: 500 µm.

TMZ is an oral alkylating agent used to treat GBM. Although it can cross the BBB, at least 50% of GBM patients do not respond to TMZ (Lee, 2016). Here, we sought to use the zPDOX model to predict TMZ sensitivity for specific GBM patients. Using our established procedure, we transiently cultured GBM tumor cells from five different patients (Table S3) and generated zPDOXs for each. The entire test, from tumor dissection to tumor imaging and measurement, was completed within 20 days following surgery (Fig. 6A).

For patient (p#)1, GBM xenografts in the zebrafish brain grew relatively slowly (produced *n*=12 samples on 3 dpi and divided into two groups). Following 3 days of TMZ treatment, the GBM xenografts almost completely disappeared, with only residual tumor mass detectable in some of the zebrafish brains. Thus, we concluded that p#1 GBM xenografts were sensitive to TMZ treatment. Consistently, 6 months after surgery, the patient displayed no detectable tumor progression or relapse following combined radiotherapy and TMZ treatment (Fig. 6B). For the other four patients, all established zPDOXs grew very robustly (*n*=12 for p#2, *n*=12 for p#3, *n*=13 for p#4 and *n*=15 for p#5). For each zPDOX, following the same TMZ treatment as we described for p#1-derived zPDOX, quantitative results showed significant inhibition of the xenograft size for p#2 and p#5, but not p#3 and p#4. Critically, 6-7 months later, according to the MRI results, p#2 and p#5 showed no indication of tumor progression or relapse. However, for p#3 and p#4, whose zPDOX only showed a weak response to TMZ treatment (some inhibition tendency, but no significant size inhibition ratio), obvious relapse was detected (6-7 months postsurgery).

By consulting postoperative medical records, we obtained the immunohistochemistry results for each patient (listed in Table S4). Previous studies (Butler et al., 2020; Lalezari et al., 2013) have found that high MGMT expression is significantly associated with poor patient survival or treatment response, resulting in TMZ treatment resistance. Indeed, pathological diagnostic results indicated a high level of MGMT expression and high percentage of KI67^+^ (also known as MKI67^+^) tumor cells (#p3 60%, #p4 50%) in the two patients with relapse. Thus, our results altogether indicated that short-term TMZ response in zPDOXs is strongly correlated with the incidence of tumor relapse in TMZ-treated GBM patients (6-7 months postsurgery), indicating the ability of zPDOXs in predicting the long-term clinical TMZ sensitivity or resistance of the residual GBM micrometastases in patients after surgery.

Although we have not yet gathered sufficient patient numbers to reach statistical significance, we provide compelling evidence from our proof-of-concept experiments that a larger clinical study is warranted. This larger study will help determine whether a short-term TMZ sensitivity assay in our zPDOX model can accurately predict long-term patient response to TMZ. Going forward, this assay will also likely be applicable to rapidly test response to other novel drugs or drug combinations.

## DISCUSSION

Orthotropic GBM animal models based on mouse and rat are valuable tools that are widely used in studies of cancer biology and drug discovery (Jacobs et al., 2011). However, these rodent-based GBM orthotropic models, whether transplanting GBM cell lines or patient-derived primary GBM cells, are generally expensive and time consuming, and studies are often limited by small experimental numbers and low reproducibility. In the past several years, zebrafish larvae have been introduced as a complementary animal model to study various human cancers (Fior and Ferreira, 2018; Fior et al., 2017; Yan et al., 2019) and have been successfully used for xeno-implantation of human GBM cells into the zebrafish larval brain (Hamilton et al., 2016; Welker et al., 2017, 2016; Zeng et al., 2017). In our previous studies, we transplanted human GBM cell lines (U87MG, U-251MG) into the zebrafish brain and found that a small compound, named TNB, could cross the zebrafish BBB and inhibit the progression of GBM cells (Zeng et al., 2017). In addition, Pudelko et al. transplanted both GBM cell lines and primary GBM cells into zebrafish blastulas, observing that GBM cells migrated from zebrafish blastomeres into zebrafish brain 24 h post-transplantation (Pudelko et al., 2018). Despite the unique advantages of the zebrafish larvae model system, including optic transparency, a naturally immature immune system and the large number of offspring produced for experimentation, the zebrafish-based GBM xenograft model is still not widely utilized in the GBM research field. This lack of utilization belies these and other documented experimental strengths. Here, we demonstrate that the zebrafish larva is a versatile model animal for GBM xeno-implantation and provide the most rigorous molecular characterization and histopathological comparison with mammalian models to date. We also demonstrate the existence of a molecularly and functionally intact BBB in zebrafish larvae, which allowed the study of interactions between infiltrating tumor cells and the BBB, and enabled us to carry out a proof-of-concept screening of BBB-penetrating drugs and predict drug sensitivity for individual GBM patients.

With our zebrafish model, we were able to monitor and document extensive intracranial invasion and tumor vascularization within GBM xenografts. Critically, we show that these characteristics vary depending on the source of implanted GBM cells and reveal the unique cellular responses that exist between and within GBM tumors. Intracranial invasion and redundant tumor vasculature are not only the most typical pathological features of human GBM, but also key factors that affect the prognosis and radiochemotherapeutic response in GBM patients (Hutchinson and Kirk, 2011; Kamb, 2005). By comparing our outcomes with previously published rodent-based GBM xenografts, we show that the zebrafish GBM xenografts can accurately phenocopy the specific histopathological characteristics of individual GBM cell lines. Moreover, for each specific GMB cell line, when dozens of zebrafish xenografts were produced, the phenotypes of the implanted xenografts were highly consistent and stable. These data demonstrated that GBM xenografts of diverse origins can consistently and robustly propagate in zebrafish brain, and that the zebrafish model system can faithfully reveal the histopathological heterogeneity of implanted GBM cells, indicating that it is a reliable animal model for biological and therapeutic studies of intracranial GBM microtumors.

Given the importance of the BBB for efficient delivery of GBM therapeutics, establishing reliable BBB models has been a top priority for GBM drug discovery. Although *in vitro* BBB models (Pardridge, 2005; Qian et al., 2017) have provided greater insight into specific aspects of GBM-associated BBB, they fail to capture the many complexities of the *in vivo* system, especially the dynamic interactions that occur between infiltrating GBM cells and host vascular cells. In the past, many studies indicated that the zebrafish was an easy and convenient model for BBB permeability assessment. By using transmission electron microscopy, Fleming et al. found that the structure of the zebrafish BBB was gradually formed from 3 dpf to 10 dpf (Fleming et al., 2013), and was similar to the mammalian BBB structure. In addition, previous studies (Li et al., 2017; Zeng et al., 2017) verified the BBB of zebrafish by using a fluorescent tracer of different molecular mass, and found that the fluorescent tracer was restricted to cerebral capillaries. However, the molecular and functional integrity of the BBB in zebrafish larvae is unclear, and its possible interaction with implanted GBM tumor cells has yet to be characterized. This information, relating to both the pathological behaviors of implanted GBM cells and the brain microenvironment of zebrafish larvae, is the fundamental basis for a reliable human GBM orthotopic xenograft model and possible application in therapeutic tests and drug screens.

Here, by using scRNA-seq and tracers of BBB function, we rigorously characterized the functional and molecular integrity of the BBB in zebrafish larvae. This work, in combination with zebrafish GBM xenografts and fluorescent BBB tracers, allowed us to establish live, *in vivo* imaging of the structural and functional changes that occur in the GBM tumor-associated BBB. Interestingly, as shown in previous mouse studies, we observed that the vascular structures in GBM xenografts are constantly altered, and, although not completely disrupted, the BBB function was gradually breached. As the BBB is a significant barrier to drugs penetrating GBM tumors, a breached BBB could potentially allow drugs access to a developing tumor. Although our model reveals that infiltrating GBM cells can breach the BBB, the magnitude of local BBB breaching from infiltrating GBM cells is likely to be insufficient to allow drug penetration in meaningful quantities. This is consistent with reports showing that brain regions experiencing tumor invasion are typically not enhanced by MRI (van Tellingen et al., 2015), even those with obvious peritumor cerebral edema. Use of our zebrafish model should help lead to a more profound structural and molecular understanding of how infiltrating GBM tumor cells dynamically interact with cerebral vasculature and the BBB and aid in the rational design of therapeutic strategies to improve the delivery of antitumor agents.

By optimizing an initial transient culture method, we have established a GBM PDOX model characterized by a high success rate for GBM propagation and rapid turnover time. We also demonstrate that the zPDOX model can accurately replicate and reveal patient-specific GBM phenotypes, making it a potentially powerful predicative tool for clinical outcomes. However, the number of tested patients is relatively small in this study, the degree to which drug efficacy in zPDOX predicts activity in human patients remains to be determined, and a future parallel study including more GBM patient samples is warranted.

Although zebrafish larvae offer many useful advantages, zPDOXs still have some inherent limitations compared with mouse-based patient-derived xenografts (PDXs). For example, human tumor cells and zebrafish have different maintained temperature, and tumor growth could be affected below 37°C. In addition, zebrafish larvae lack the human immune environment. Recent studies have established PDX models in humanized mice, which offer the added advantage over standard PDX models of being able to assay patient-specific tumor response in the mouse brain, and could better retain the biological and molecular features of human tumors (Welker et al., 2016; Zeng et al., 2017).

Overall, we have now established an alternative PDOX model using zebrafish larvae. As the number of GBM cells implanted into a single zebrafish is relatively small, dozens of xenografts can be established from a very small piece of patient GBM tissue. This allows for analysis of several samples per patient, which increases the chances of detecting internal clonal heterogeneity of the primary GBM, which can impact clinical prognosis and treatment plans. Given these advantages of high success rate, speed and high sample numbers per patient, the zPDOX model will be useful for rapidly evaluating drug response for clinical decision making in precision medicine.

## MATERIALS AND METHODS

### Transgenic zebrafish maintenance and establishment

Lines used in this study included Tg(*kdrl*:EGFP)^s843^, Tg(*kdrl*:mCherry)^uto2^ and Nacre mutant (*mitfa*w2, skin-pigmentation mutation). To avoid pigmentation, we established a *kdrl*:EGFP Nacre line by direct outcross and incross of *kdrl*:EGFP with Nacre fish. Zebrafish were maintained at 28.5°C with a 14-h light and 10-h dark cycle. Fish water was changed daily, and larvae that were more than 7 days old were fed twice a day with grinded brine shrimp. All zebrafish experiments were conducted in accordance with the guidelines of the Animal Care and Use Committee of Sichuan University (Chengdu, Sichuan, China) and approved by the institutional review board of the Medical Faculty at the West China Hospital, Sichuan University.

### Glioma cell lines and culture

Glioma cell lines U87MG, U-251MG, GL261 and C6 were purchased from American Type Culture Collection. These glioma cell lines were cultured in Dulbecco's modified Eagle medium (DMEM) (Gibco), containing 10% fetal bovine serum (FBS) (Gibco) and 1:100 penicillin/streptomycin (Invitrogen) at 37°C with 5% CO_2_. Adherent cells were detached and dissociated with 0.025% trypsin (Gibco) for 2 min. The GSCs (BNI-21, BNI-23) were cultured in Stem Cell Medium [neurobasal medium (Gibco) containing 2% B27 (Gibco), 10 ng/ml bFGF (R&D Systems), 20 ng/ml EGF (R&D Systems), Glutamax (Invitrogen), sodium pyruvate (Invitrogen) and antibiotics (Invitrogen)]. All human cell lines used in this work were authenticated just prior to use. To establish stable mCherry-expressing glioma cell lines, wild-type glioma cells were infected with lentivirus containing a cytomegalovirus (CMV) driving mCherry gene. Fluorescence-activated cell sorting (Aria, BD Biosciences) was applied to sort the cells with same fluorescent intensity 48 h postinfection. The stable mCherry-expressing cells were cultured in normal conditions.

### Zebrafish xenograft injection

We tested to inject GBM cells into zebrafish brain at 2, 3, 4 and 5 dpf, and compared the success rate and growth of implanted GBM xenografts. We found that injecting GBM cells at 3 dpf yielded a higher success rate and allowed a relatively long observation window. The 5×10^6^ mCherry-expressing glioma cells (U87MG, U-251MG, Gl261 and C6, ∼100 cells per fish) were suspended in 50 μl basic DMEM and loaded into a borosilicate glass needle pulled by a Flaming/Brown micropipette puller (Narishige, PN-30). Zebrafish larvae at 3 dpf were anesthetized with 0.2 mg/ml Tricaine (Sigma-Aldrich) before tumor injection. To establish the experimental zebrafish glioma model, a 3-5 nl suspension containing ∼100 cells (Fig. S1) was injected into the TeO of *kdrl*:EGFP Nacre zebrafish larvae (Fig. 1B). Recipient zebrafish larvae injected with tumor cells were maintained in fish water containing the antibiotics at 33°C. Twenty-four hours postinjection, zebrafish with cellular debris or cells in the ventricle were discarded. Around 100 tumor cells in midbrain parenchyma of zebrafish brain were regarded as successful injection of zebrafish, and these injected zebrafish were randomly collected to each group separately until the end of the experiment.

### scRNA-seq for GBM cells implanted in zebrafish brain

We conducted scRNA-seq to reveal transcriptome changes of GBM cells after implanting in zebrafish brain. The experimental strategy is outlined in Fig. 2A. For GBM cells, zebrafish were injected with GBM-U-251MG-mCherry cells and allowed to form widespread, tumor invasions for 4 days. At 4 dpi, the brains of the zebrafish larva were isolated using tweezers. Under the fluorescence stereomicroscope, the core area and tumor invasion area of the glioma xenografts from the zebrafish brain were divided into two dishes, and completely disaggregated using 1 mg/ml papain and 1 mg/ml DNase at 37°C for 30 min. After digestion, a single-cell suspension was made by resuspending the cells in DMEM with 2% FBS. Simultaneously, GBM-U-251MG-mCherry cells maintained in culture were trypsinized and resuspended in DMEM with 2% FBS. The mCherry-positive single cells from the cell suspension were sucked up into a vial containing lysis buffer by a borosilicate glass needle under a fluorescence micromanipulation system (TransferMan NK 2, Eppendorf). Four tumor cells from the *in vitro* culture system, nine tumor cells from the tumor core area and eight tumor cells from the tumor invasion area were sucked up to prepare the scRNA-seq library. The scRNA-seq library was prepared according to the Smart-seq2 protocol (Picelli et al., 2013). Samples were sequenced using a HiSeq2500 (Illumina) with ∼20 million reads per sample, using 150-bp paired-end reads. The RNA-seq reads were aligned to the reference genome (GRCh39/GRCz10) by STAR_2.6.0a. Transcript abundance was normalized and measured in fragments per kb of exon per million fragments mapped (FPKM). Differential gene expression was analyzed by DESeq2. Genes with false discovery rate≤0.05 were counted as differentially expressed genes.

### PCA and GO analysis

We performed a PCA analysis of the gene expression values (FPKM) for all samples. In the PCA diagram, the samples between the groups should be dispersed, and the samples in the group should be brought together. In order to reflect the difference in gene expression between samples, we used hierarchical clustering to cluster the gene expression values and homogenize the lines of expression data. Genes or samples with similar expression patterns in the heat map will be clustered together, and the color in each square reflects the expression of the gene. In addition, we used the GO database, which contained biological processes and cellular components. GO function enrichment uses *P*<0.05 as the threshold for significant enrichment. All heatmap analyses were generated using the software Gene-E.

### scRNA-seq for endothelial cells of zebrafish

To detect BBB-related gene expression by ECs in zebrafish brain and the body trunk, the *kdrl*:EGFP zebrafish (5 dpf, *n*=5) were used to obtain ECs. Under the stereomicroscope, the brains of the zebrafish larva were isolated using tweezers. The brain and body trunk of zebrafish larvae were divided into two dishes and completely disaggregated. Under the fluorescence stereomicroscope, a single GFP-expressing EC from cell suspension was sucked up by a borosilicate glass needle. Nine cerebral ECs and three body ECs were sucked up to prepare the scRNA-seq library. The scRNA-seq library was prepared and data analyses performed as above.

### GSEA

The BBB gene set contains 20 tight junction-related genes, 25 CLDN genes, 407 solute carrier transporter genes and 53 ATP-binding cassette transporter genes. The zebrafish gene symbols were mapped to their human orthologs using the DIOPT tool (http://www.flyrnai.org/cgi-bin/DRSC_orthologs.pl). From the zebrafish RNA-seq data set, we created a BBB-related list of genes either upregulated or downregulated in the zebrafish cerebral ECs versus trunk ECs, and these were input into GSEA as GCT files. In addition, we used heatmaps to show the BBB-related gene expression patterns between cerebral and body ECs.

### Pericardium microinjection of Dextran Blue, DAPI and NaF

In order to *in vivo* monitor the permeability of cerebral and body vessels in zebrafish, NaF (376 Da; Sigma-Aldrich), DAPI (350 Da; Sigma-Aldrich) and Dextran Blue (10,000 Da; Invitrogen) were dissolved in PBS to final concentrations of 2 mg/ml, 1 mg/ml and 5 mg/ml, respectively. Approximately 5-10 nl of tracers were injected into each common cardinal vein using glass capillary needles. Injected zebrafish were washed twice with fresh fish water, and confocal live images were taken within 1-2 h after the tracer injection.

### Human tissue processing and primary culture

All samples used for zPDOX establishment were obtained from patients who were diagnosed with GBM by enhanced MRI and pathological diagnosis at West China Hospital (Table S3). Written informed consent was obtained from all patients. Our study was approved by the institutional review board and ethics committee of Sichuan University. The fresh GBM tissues were digested into single cells by Accutase (Sigma-Aldrich) for 30 min at 37°C, and further washed with cold PBS (three times) with gentle vortexing. The single-cell suspensions were collected and passed through a 70-μm strainer (Millipore). These human primary GBM cells were cultured using three cell culture methods: organoid culture, neurosphere culture and adherent culture (Hubert et al., 2016). For 3D organoid culture, red blood cells were removed by brief hypotonic lysis, and GBM cells were counted for cell number and viability using Trypan Blue. Around 105 cells were mixed with 20 μl Matrigel (Corning), and dropped into a 24-well plate. After solidifying (15 min, 37°C), the gels were overlaid with 500 μl Stem Cell Medium. For neurosphere culture (Jacob et al., 2020), we resuspended the human GBM cells in 10-20 ml neurobasal medium plus all additives and incubated them at 37°C overnight. The next day, we lysed red blood cells and change the medium (Stem Cell Medium). The GBM stem cells were cultured for 1 week to form neurospheres. For adherent culture, human GBM cells were cultured in (DMEM/F12) (Gibco), containing 2% B27 (Gibco), 10 ng/ml bFGF (R&D Systems), 20 ng/ml EGF (R&D Systems), 5% FBS (Gibco) and 1:100 penicillin/streptomycin (Invitrogen).

### Establishment of zPDOXs

To compare three cell culture methods for the establishment of zPDOX models, we injected human primary GBM cells into zebrafish brain using the three cell culture methods, organoid culture, neurosphere culture and adherent culture. Before injection, the cultured human primary GBM cells were digested into single-cell suspensions. These cells were labeled *in vitro* using the carboxyfluorescein succinimidyl ester (CFSE) vibrant cell labeling solution (Life Technologies), at a final concentration of 1 mmol/l in 0.1% bovine serum albumin in DMEM for 30 min at 37C in a 5% CO_2_ atmosphere, as previously reported. The CFSE-labeled GBM cells were injected into zebrafish brain as described above. The comparison of human GBM engraftment zebrafish brain at 3 dpi was identified by a Zeiss LSM 880 confocal microscope.

### Zebrafish xenograft drug administration

In order to find a convenient and quick zebrafish administration method for glioma xenografts, fluorescent NaF tracer (10 μM) and DAPI (1 μM) were directly added to fish water containing zebrafish at 5 dpf, and the zebrafish were incubated for 2 h (Fig. 4A). Fluorescent staining of zebrafish was detected by the Zeiss LSM 880 confocal microscope and was used to monitor the delivery route of small-molecule chemicals into the brain. Six anticancer drugs, associated with treatment of glioma, were chosen from the Selleck Anti-cancer Compound Library. These six anticancer drugs – TMZ (500 μM), vincristine (10nM-20 nM), doxorubicin (1 μM-2 μM), DEX (10 μM), rapamycin (25 μM) and gemcitabine (20 μM) – were tested *in vivo* and *in vitro*. Concentrations of these drugs were determined by zebrafish maximum-tolerated concentration and effective tumor concentrations tested *in vitro*. Zebrafish xenografts with the same tumor size were randomly distributed in the treatment group and control group on 2 dpi. Drugs were added directly to fish water, and the drug was changed daily for the next 3 days. The corresponding concentration of dimethyl sulfoxide was added to the control group. After 3 days of treatment, the regression of the tumor in zebrafish xenografts was identified under the fluorescence stereomicroscope. Tumor size was determined by quantifying the 2D image area using ImageJ and multiplying by the average mCherry fluorescence intensity.

### zPDOX TMZ administration details

After a week of culture, human primary GBM cells were infected with lentivirus containing a CMV driving mCherry gene. The stably expressed mCherry GBM cells were injected into zebrafish brain as described above. The standard chemotherapy drug for high-grade GBM, TMZ, was used to test whether a short-term response to TMZ treatment in zPDOX would anticipate a delay in relapse in the matching patients. TMZ was dosed and administered as described above.

### Hematoxylin and Eosin (H&E) staining

The zebrafish xenograft was fixed in 4% paraformaldehyde, embedded in paraffin and sectioned at 3 μm thickness. Sections were stained with H&E for routine histopathological analysis.

### EdU labeling and MTT assay

For EdU labeling, we added the EdU (500 mM) to the zebrafish water at 2 dpi, fixed the sample after 12 h and used the Click-iT EdU Kit (Invitrogen) to label the proliferating cells according to the manufacturer's instructions. MTT assay was performed according to the manufacturer's instructions (Solarbio, M1020).

### Immunofluorescence

The *in vitro* cultured human primary GBM cells were fixed in 4% formaldehyde. Primary antibodies used were anti-human NES (rabbit, ab105389, Abcam) and anti-human GFAP (mouse, ab10062, Abcam), at 1:100. Secondary antibodies used were Alexa Fluor goat anti-mouse 488 (A-10667, Thermo Fisher Scientific; 1:400) and Alexa Fluor goat anti-rabbit 594 (A-11012, Thermo Fisher Scientific; 1:400). Nuclei were counterstained with DAPI.

### Live zebrafish imaging and qualification

For the entire zebrafish images, the anesthetized embryos were mounted in 0.08% methyl cellulose. Both fluorescent and bright-field images were taken by a Zeiss SteREO Discovery V20 stereomicroscope (Carl Zeiss, Germany). The whole brain of zebrafish was imaged with a Zeiss LSM 880 confocal microscope with Airyscan (Carl Zeiss) under a 20× lens with a total depth of 100 μm. For whole-brain *z*-stack images, maximum projection images were created from 100 μm stacks (10 μm per confocal slice) using ZEN blue software (Carl Zeiss). Tumor size was determined by quantifying the 3D image area of mCherry fluorescence using Imaris software (Biplane) (Fig. S6). For digital living image of tumor proliferation and angiogenesis, zebrafish embryos were mounted in low-melting-point (LMP) agarose (1.5%, w/v) at the bottom of a 29 mm glass-bottom dish and covered with fish water at 28°C. The micrograph images were taken with the Zeiss LSM 880 confocal microscope with Airyscan under a 20× lens with a total depth of 200 μm. To calculate the infiltration distance of tumor cells from core margins, the infiltration distance of tumor cells was measured by the ZEN blue software, and the digital tumor images were 3D reconstructed by Imaris. For detection of the BBB function in zebrafish larvae, the maximum projection images were analyzed by Profile in ZEN blue, which showed the fluorescence intensity of vessels and Dextran Blue.

### Clinical human GBM data

All patients in our study received the standard concurrent chemoradiotherapy after operation. The clinical data and specific chemoradiotherapy regimen of each patient are listed in Table S3. The surgeon resected the tumor during the operation, and no tumor residue was found by postoperative MRI. The recurrence of GBM was confirmed by enhanced MRI at 6-7 months after the operation. The enhanced MRI images were assessed by two radiologists independently, and disagreements were resolved by discussion or the involvement of the third radiologist. All clinical investigation was conducted according to the principles expressed in the Declaration of Helsinki.

### Statistical analysis

In all experiments, zebrafish were randomly assigned to experimental groups. GraphPad Prism software was used for statistical analysis. All datasets were challenged by a normality test. Datasets with a normal distribution were analyzed by unpaired two-tailed Student's *t*-test. *P*<0.05 was considered statistically significant.

## Supplementary Material

Supplementary information
