# Supplementary material for: Clinically relevant orthotopic xenograft models of patient-derived glioblastoma in zebrafish
Source: Dis Model Mech. 2022 Apr 26;15(4):dmm049109. doi: 10.1242/dmm.049109 (PMC9066514; doi:10.1242/dmm.049109)
Supplement: Supplementary information [file dmm-15-049109-s1.pdf]

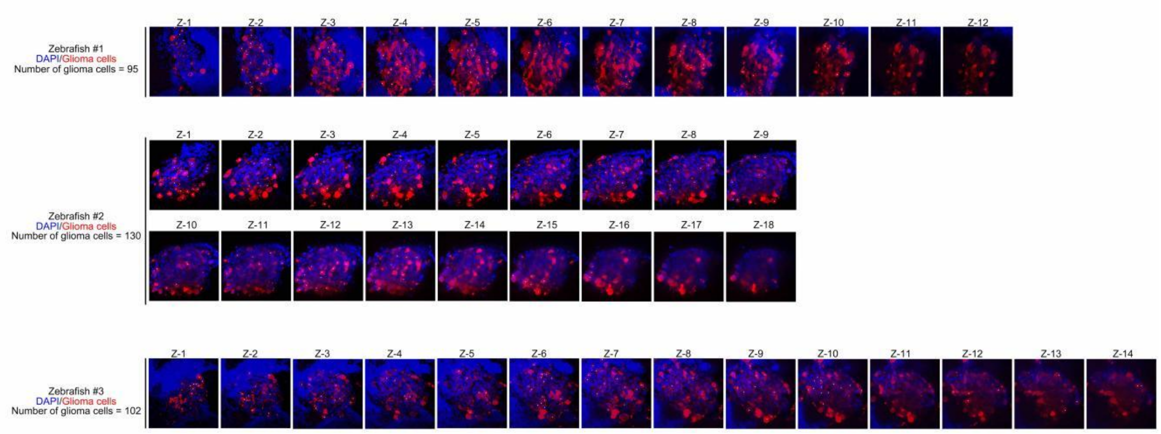

Fig. S1. The whole brain confocal imaging shows three zebrafish Gl261-mCherry xenografts. DAPI (blue) and mCherry (red) double positive were marked (white dots) and counted at 2dpi. Scale bar, 50μm.

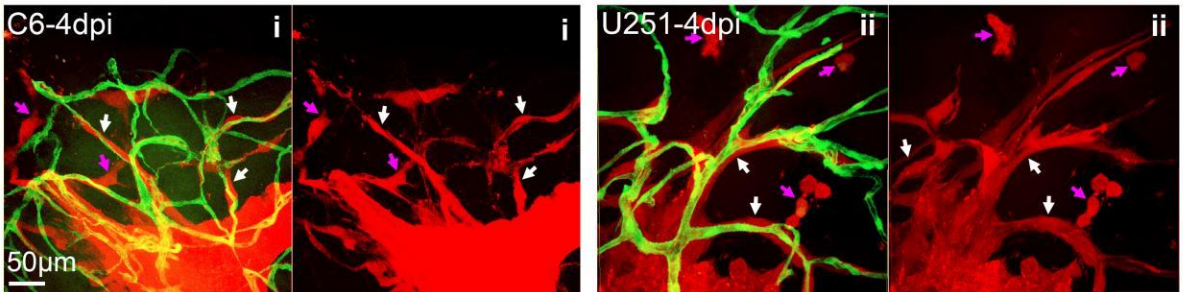

Fig. S2. Zebrafish orthotopic xenografts reveal the specific in vivo histopathological features of GBM tumor cell lines. Image i and ii respectively show the infiltration of rat C6 xenografts (4dpi) and human U251MG xenografts (4dpi) in the zebrafish brain (kdrl:eGFP). White arrows indicate the tumor cells infiltrating along cerebral vessels and magenta arrows indicate the tumor cells invading intraparenchymal. Scale bar, 50μm.

Disease Models & Mechanisms • Supplementary information

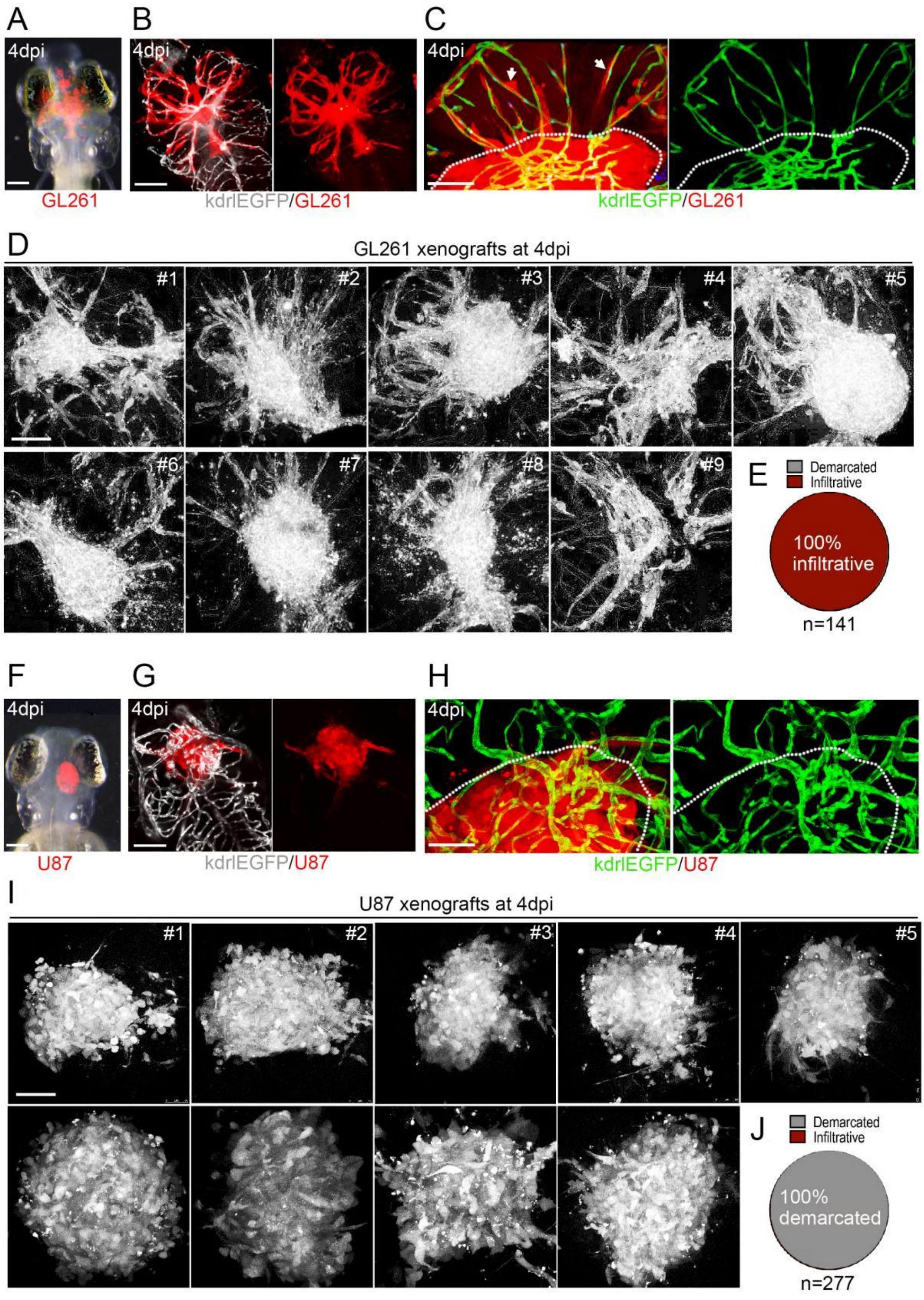

Disease Models & Mechanisms • Supplementary information

Fig. S3. Zebrafish orthotopic xenografts reflect the intra-tumor homogeneity within GBM tumor cell lines. (A, B) Fluorescent stereo microscope and confocal imaging show the whole brain infiltration of GL261 cells at 4dpi in the zebrafish brain. (C) Confocal imaging shows the extensive infiltrating of GL261 cells (white arrows) along the cerebral capillaries (kdrl:EGFP), dotted line indicating the xenograft edge. (D, E) Confocal imaging of 9 typical GL261 xenografts shows the stable and homogenous infiltrative growing pattern in the zebrafish brain at 4dpi. Totally n=141 GL261 xenografts were evaluated. (F, G) Fluorescent stereo microscope and confocal imaging show the demarcated U87MG xenograft at 4dpi in the zebrafish brain. (H) Confocal imaging shows the tumor angiogenesis within the U87MG xenograft, dotted line indicating the xenograft edge. (I, J) Confocal imaging of 9 typical U87MG xenografts shows the stable and homogenous demarcated growing pattern in the zebrafish brain at 4dpi. Totally n=277 U87MG xenografts were evaluated. Scale bars, 100μm.

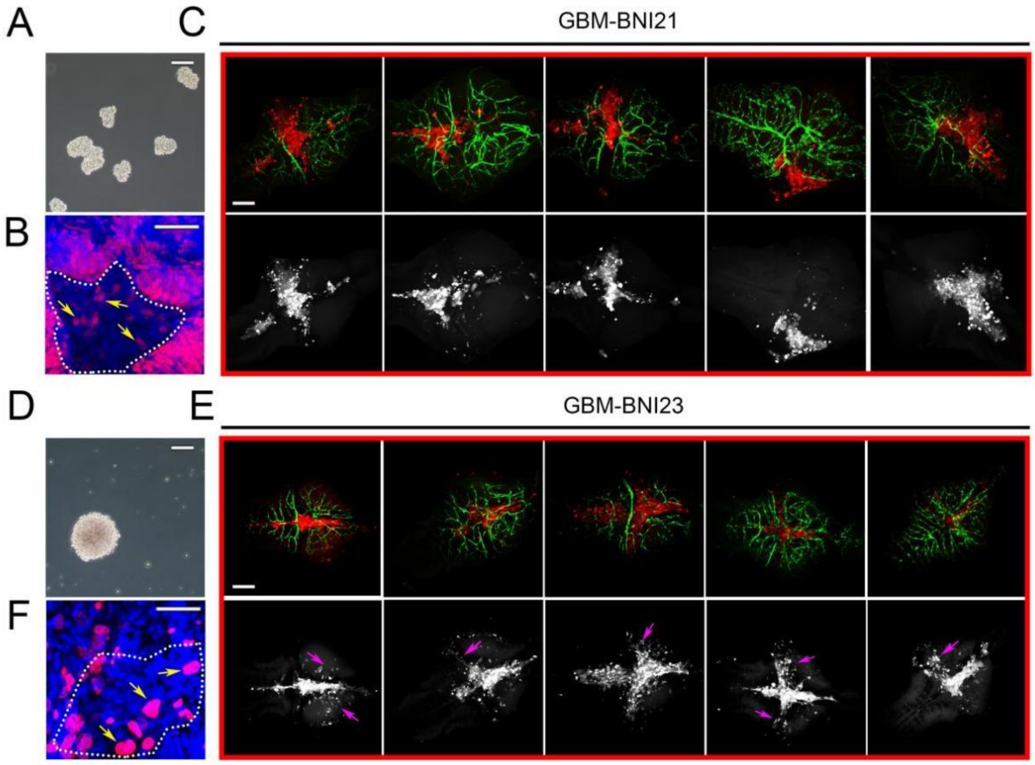

Fig. S4. Zebrafish orthotopic xenografts can be efficiently established using patient derived GSCs. (A, D) Bright-field of in vitro culturing patient derived GSCs (BNI21, BNI23). (B, F) The tumor confocal imaging shows zebrafish xenografts of BNI21 and BNI23 at 5 dpi. DAPI (blue) and EdU (red) double staining was applied at 5 dpi. EdU positive tumor cells (yellow arrows). (C, E) At 5dpi, BNI23 showed a typical infiltrative growth pattern of GBM in the zebrafish brain. Scale bars, 100μm.

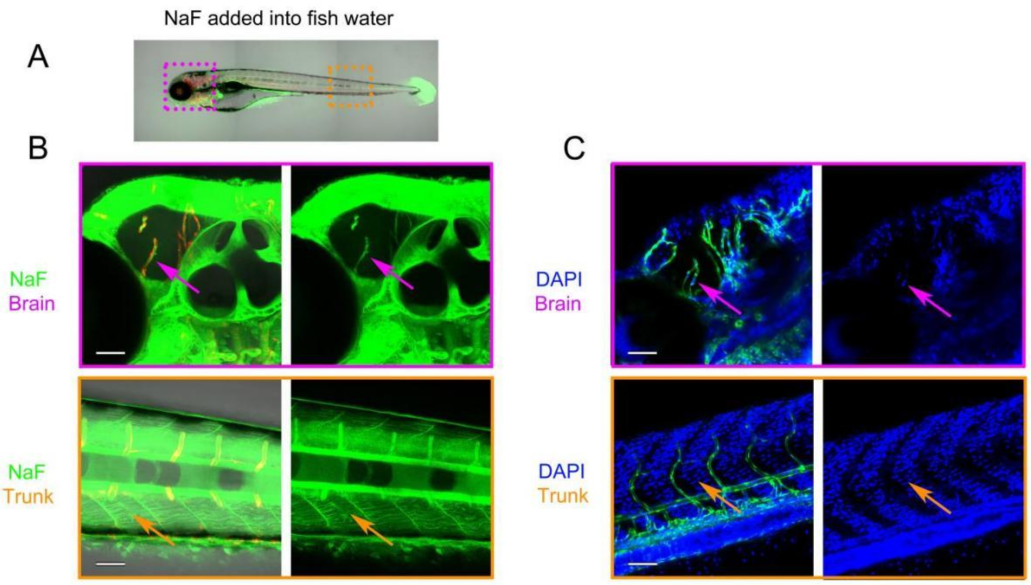

Fig. S5. Florescent BBB tracer in the zebrafish model system. (A) Schematic showing the BBB tracer NaF penetrating zebrafish tissue (5dpf) from culturing water, areas in the dotted boxes are magnified in B. (B) NaF (376 MW) (10μM) was added directly into fish water 2 hours before imaging. Within the brain, NaF is restricted in cerebral vessels (Magenta arrows), but not freely diffusing in the parenchyma. Within the trunk, NaF is diffused into the muscle fibers (orange arrows). (C) DAPI (2μg/ml) was added directly into fish water 2 hours before imaging. Within the brain, the cerebral endothelial nuclei are labeled by DAPI (Magenta arrows), while the nuclei of parenchymal cells outside of cerebral vessels are not labeled (purple arrow). Within the trunk, the nuclei of muscle fibers are labeled by DAPI (orange arrow). Scale bars, 100μm.

Disease Models & Mechanisms • Supplementary information

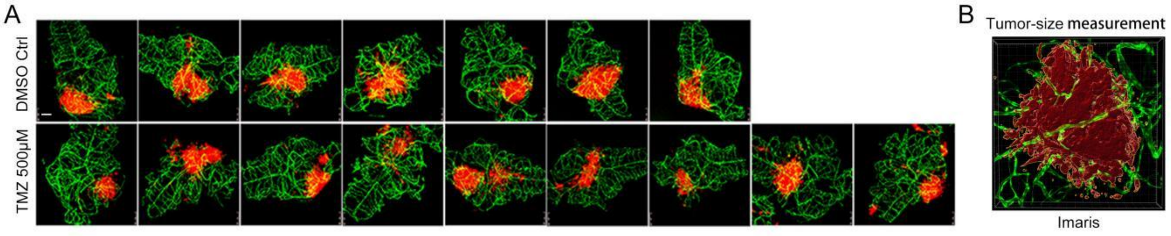

Fig. S6. GBM xenografts show sensitivity to TMZ. (A) images show the U87MG xenografts (red) in the dissected zebrafish brains (kdrl:eGFP) after treated by temozolomide (TMZ, 500μM) for 3 days. (B) Representative image shows the measurement of xenograft by Imaris. Scale bars, 100μm.

Disease Models & Mechanisms • Supplementary information

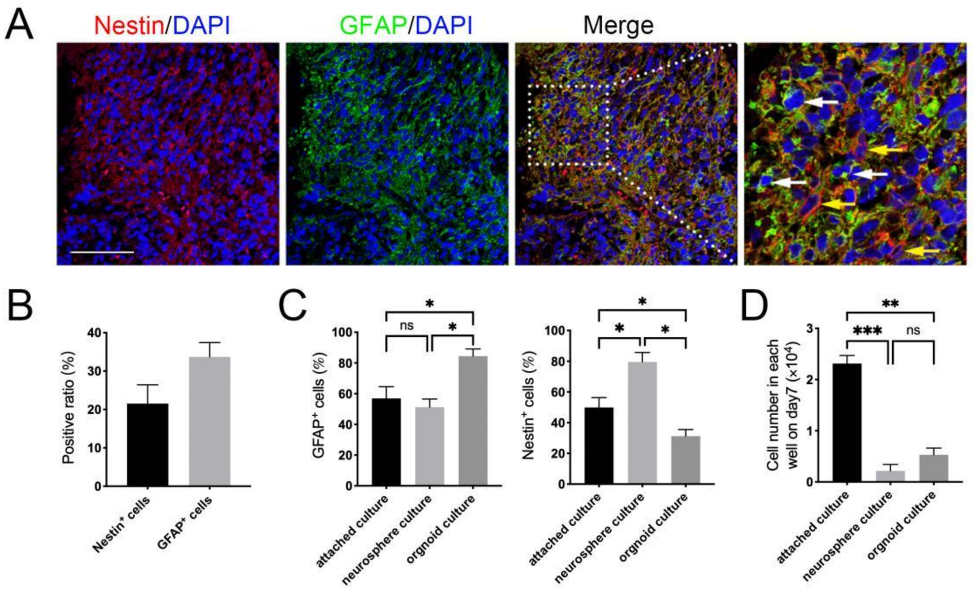

Fig. S7. Staining of resected GBM samples. (A) Patient GBM tissue stained for Nestin$^+$ (red, yellow arrows) and GFAP$^+$ (green, white arrows). (B) Quantification of Nestin$^+$ and GFAP+ ratio. (C) Graph shows the percentage of Nestin$^+$ and GFAP$^+$ cells (Passage 1) in the different transient culture systems (Attached culture, Neuroshpere culture and Organoid culture), Nestin$^+$ and GFAP$^+$ cells were determined by IF staining. (D) Graph shows the cell numbers that can be enriched in passage1 on day7 from different transient culture systems. GBM#109 was tested in this experiment. Scale bars, 100μm.

Disease Models & Mechanisms • Supplementary information

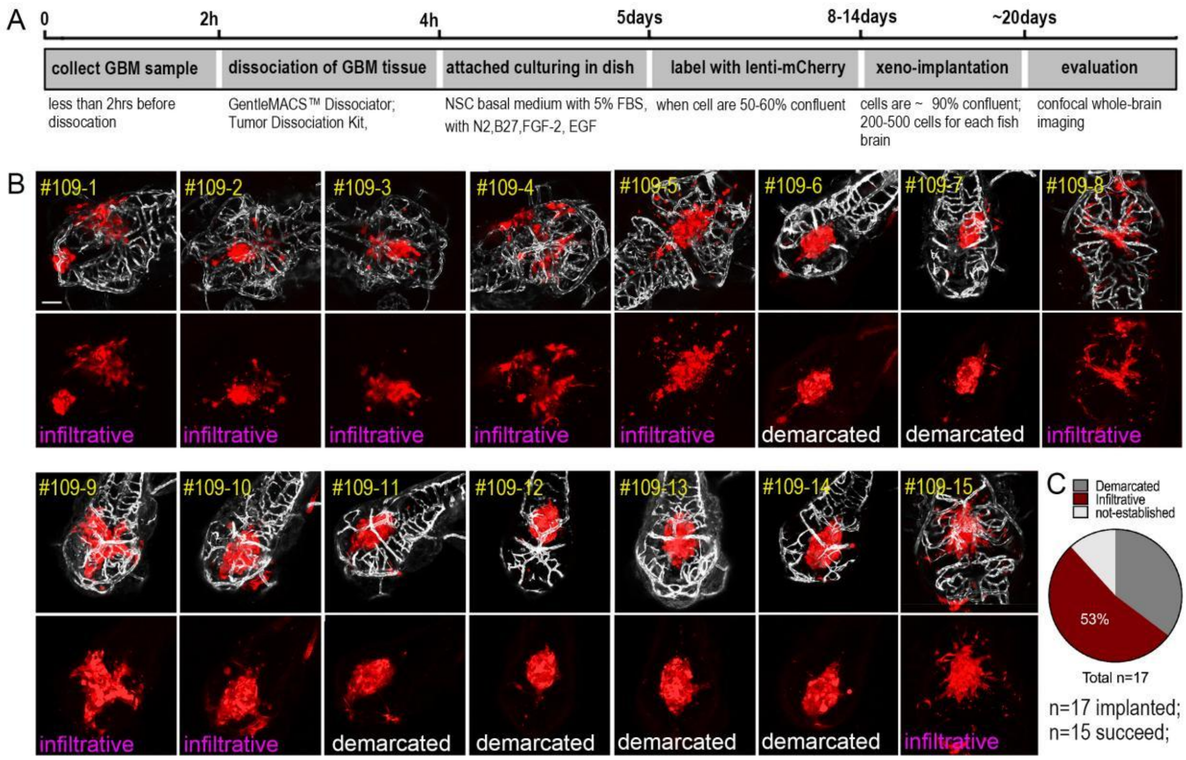

Fig. S8. Zebrafish patient-derived orthotopic xenografts reveal the heterogeneity of GBM patient. (A) Timeline shows the procedure of transient *in vitro* culture and implantation of patient derived GBM cells. The culturing time for passage1 (P1) GBM cells varied from 5days to 2weeks, depending on individual patients. (B) Images show the phenotypes of individual patient derived xenografts (n=15, GBM#109, 4dpi) in zebrafish brain (kdrl:eGFP, gray channel), indicating the intra-heterogeneity of primary GBM cells (P1). (C) Diagram shows the percentage of infiltrative and demarcated xenografts in the zebrafish brain, xenograft has more than 5 detached invading cell clusters from the tumor core was defined as infiltrative.

Disease Models & Mechanisms • Supplementary information

Table S1. Differential expression gene list in zebrafish xenograft versus in vitro cultured cells

Click here to download Table S1

Table S2. The 506 genes of BBB gene set were enriched in zebrafish cerebral endothelial cells

Click here to download Table S2

Table S3. Summary of the clinical data of GBM patients and experimental results derived from primary cultured GBM cells

Click here to download Table S3

Table S4. Postoperative pathological diagnostic results of patients

Click here to download Table S4
